# Age and Hydration of Competing Horses Influence the Outcome of Elite 160 km Endurance Rides

**DOI:** 10.3389/fvets.2021.668650

**Published:** 2021-05-14

**Authors:** Lena Bollinger, Alexander Bartel, Alina Küper, Corinna Weber, Heidrun Gehlen

**Affiliations:** ^1^Equine Clinic, Internal Medicine, Freie Universität Berlin, Berlin, Germany; ^2^Institute for Veterinary Epidemiology and Biostatistics, Freie Universität Berlin, Berlin, Germany; ^3^Laboklin Veterinary Laboratory Diagnostics, Bad Kissingen, Germany

**Keywords:** endurance horse, endurance riding, World Championship, long-distance riding, infusion, risk factor, elimination, age requirements equestrian sports

## Abstract

High elimination rates and the concern for horse welfare are important issues in endurance riding. An improved understanding of the causes of elimination could increase completion rates in this sport. We have identified pre-ride risk factors that allow an assessment of potential elimination before the ride. A longitudinal cohort study was performed among 49 healthy horses competing in the 160 km endurance ride at the 2016 World Championship of Endurance Riding in Samorin/Slovakia. Blood samples were drawn before the ride. For statistical evaluation, horses were categorized in three groups: finishers, lame and metabolically eliminated horses. Risk factors were calculated for each group using multinomial logistic regression. A 1% increase in hematocrit levels was associated with a higher OR for elimination (lameness: OR 1.26, *p* = 0.017; metabolic: OR 1.34, *p* = 0.010). Furthermore, increased potassium values correlated negatively with the race outcome. For a 1 mmol/l increase in potassium, the lameness OR was 4.21, *p* = 0.039 and metabolic OR was 1.15, *p* = 0.848. Eight-year-old horses had a 100% elimination rate and survival analyses showed a significantly higher hazard for elimination (*p* = 0.025). We thus conclude that age and hydration affect the outcome of elite endurance rides. Further investigation of age as a risk factor seems to be clinically relevant and adjustments of FEI qualification modes may be appropriate.

## Introduction

The demanding sport of endurance riding has come under scrutiny due to questions of horse welfare and lethal injuries in the past decade (1–5). After the disastrous World Equestrian Games in Tryon/USA in 2018, the World Equestrian Federation (FEI) began seeking solutions to improve horse welfare as well as the public reputation of the sport (6). Elimination rates at international rides have remained consistently high over the last years ranging between 50 and 80% while the speeds of the races have increased (5, 7).

Following physical exercise, specific changes in metabolic reactions occur in athletic horse leading to several changes in the body, mainly in the circulatory, respiratory, endocrine, and neuromuscular systems (8). Changes taking place in these systems simultaneously and in an integrated manner are aimed at maintaining homeostasis in the body (9). However, long-term physical exertion may result in disturbances of homeostasis, such as energy depletion and changes in fluids, electrolytes and acid-base balance, with negative consequences for health status and performance of horse.

In 2009, Jami Whiting (10) published an article outlining the special physiological characteristics of endurance horses after elimination. The author stated that many well-trained endurance horses have low resting hematocrit levels compared to horses of other disciplines. Therefore, even mild hematocrit elevations point to a severe problem (4, 11). This supports the argument of Fielding et al. who observed only small changes in laboratory parameters in 30 endurance horses treated for colic following a 160 km ride (12). In addition, Whiting addressed the importance of sufficient hydration but did not further investigate water loss and associated transportation stress (10). Despite the lack of any scientific foundation, infusions before the race are a common practice at international rides as riders hope to increase water reserves in order to improve performance.

Stull and Rodiek and Padalino et al. performed research on transportation stress and measured correlating changes in different blood parameters (13, 14). Hematocrite (HCT) and creatine kinase (CK) increased during transportation. At the same time, white blood cells (WBC) showed a progressive increase. They suspected these responses to influence the energy availability for athletic performance following a lengthy transportation of horses (7). Horses of the Padalino study showed increased heart and respiratory rates, capillar refill time and neutrophil count (10).

Other risk factors for elimination may include venue- and environment-related aspects (15), riding strategy (16), or high running speeds (17). Fielding et al. found breeds like Appaloosas or Quarter horses to have a higher risk of elimination compared to Arabians and crossbreeds. They considered this might be due to a higher body mass index (18). In another retrospective evaluation, it was suspected that age plays a role, but data for 160 km rides was only available for metabolic eliminations without any age relation (19). Among endurance riders, the view is common that horses reach their performance peak between 11 and 13 years of age. This is supported by a study comparing the metabolism of six-year-old horses with the metabolism of horses with a mean age of 10.2 ± 2.0 years (20). Among humans, a recent study defined the best finishing age for ultra-marathon runners between 35 and 45 years (21). Taking this into consideration—along with high speeds as well as horse fatalities—the questions arise whether most horses even reach their best performance age and how long horses can actually endure in this sport (22, 23). However, there is no valid data on how many horses fail to reach the actively competing age of 10 or older and how long they perform in endurance riding.

During the statistical evaluation, only few of the past publications related to endurance riding used confounder adjustment (17, 18). Special focus must be placed on the non-confounder study of Trigo et al. which found HCT, plasma protein and CK to be valid parameters for risk assessment (24). Nevertheless, as elimination risk seems to be the result of multiple factors, confounder adjustment is indispensable.

To our knowledge, there have not been any scholarly publications using data of international championships, which provide a study population of the world's best horses. This study addresses the questions (25) whether lameness and metabolic status are linked by distinguishing between the two groups compared to finishers and whether certain pre-ride parameters point to a later elimination. It seeks to provide a better understanding of the risk factors leading to high elimination rates and horse fatalities. Furthermore, the study offers recommendations for adapting FEI rules to improve overall horse welfare.

## Materials and Methods

During the 2016 World Championship of Endurance Riding in Samorin/Slovakia, blood samples were taken from 49 participating horses.

Participation in our study was voluntary and free of charge for the participants. Information on the study was sent to all starting national federations in advance *via* email, which forwarded the information to their athletes. In addition, a social media post was placed to reach as many participants as possible. Owner-informed consent was obtained before any animal examination and sampling. The study was performed in accordance with EU Directive 2010/63/EU, the German Animal Welfare Legislation and the guidelines of the Freie Universität Berlin on the protection of animals used for scientific purposes. Diagnostic blood collection is a common pre-ride procedure in elite equine athletes and constitutes a standard clinical veterinary practice. According to the mentioned legal regulations, the study cannot legally beclassified as an animal experiment. Sampling therefore does not need any separate ethical approval, according to German legislation, as it is a common part of performance monitoring in endurance riding in accordance with good veterinary practice. The blood sampling was performed by trained veterinarians, while the owners or legal representatives (FEI licensed and accredited trainers) were present.

The horses included in the study started for a total of 23 nations (Algeria, Australia, Bahrain, Belgium, Chile, Colombia, Croatia, Denmark, Ecuador, Germany, United Kingdom, Hungary, Italy, Malaysia, Netherlands, Norway, Portugal, Russia, South Africa, Sweden, Thailand, Uruguay, USA). Relevant information of the horses' signalment (sex, age, breed, color) was taken from the FEI Horse Database. According to the FEI Data, 40 horses were purebred or crossbred Arabians. Four horses were listed as “other” and five horses were referred to as “unknown” in the database (Table 1).

**Table 1:** Distributional characteristics of the study population and summary statistics of blood values.

		**Finished**	**Lameness**	**Metabolic**	**Missing**
***n***		18	20	11	
**Sex (%)**					
Male		8 (44.4)	11 (55.0)	7 (63.6)	0%
Female		10 (55.6)	9 (45.0)	4 (36.4)	
**Age [median (range)]**	Years	12.0 (9.0, 16.0)	11.0 (8.0, 17.0)	11.0 (8.0, 17.0)	0%
**Weight [median (range)]**	kg	405.0 (345.0, 440.0)	402.0 (350.0, 450.0)	408.0 (380.0, 450.0)	0%
**Time between blood sampling and race [mean (SD)]**	Days	2.8 (1.1)	2.7 (0.9)	3.0 (0.8)	0%
**Breed (%)**					
Purebred Arabians		8 (44.4)	10 (50.0)	6 (54.5)	0%
Anglo-Arabians		2 (11.1)	5 (25.0)	0 (0.0)	
Shagya-Arabians		2 (11.1)	2 (10.0)	0 (0.0)	
Arabian Partbreds		1 (5.6)	0 (0.0)	3 (27.3)	
Other		2 (11.1)	1 (5.0)	1 (9.1)	
Unknown		3 (16.7)	2 (10.0)	1 (9.1)	
**Infusion (%)**					
No		4 (26.7)	13 (65.0)	8 (72.7)	6.1%
Yes		11 (73.3)	7 (35.0)	3 (27.3)	
**Median (range)**					
**RBC**	10^6^/mm3	6.9 (5.8, 8.6)	8.0 (6.2, 11.0)	7.7 (5.8, 9.4)	0%
**HGB**	g/dl	12.1 (9.8, 14.2)	13.1 (10.8, 17.7)	12.9 (9.6, 15.7)	0%
**HCT**	%	32.8 (26.7, 39.5)	37.8 (28.8, 50.0)	37.0 (28.2, 45.4)	0%
**MCV**	μm3	47.0 (45.0, 52.0)	48.0 (45.0, 51.0)	48.0 (46.0, 50.0)	0%
**MCH**	pg	16.9 (15.2, 21.1)	16.8 (15.4, 19.0)	16.7 (14.4, 19.1)	0%
**MCHC**	g/dl	35.6 (33.9, 41.5)	35.0 (33.2, 41.3)	34.6 (30.7, 39.7)	0%
**RDW**	%	17.0 (16.1, 18.1)	17.0 (15.8, 18.2)	17.5 (16.3, 17.8)	0%
**PLT**	103/mm3	173.0 (120.0, 213.0)	185.5 (130.0, 214.0)	182.0 (157.0, 217.0)	0%
**MPV**	μm3	5.6 (5.1, 6.1)	5.6 (5.2, 6.4)	5.5 (5.0, 5.8)	0%
**WBC**	103/mm3	8.0 (5.6, 12.3)	9.1 (5.6, 14.3)	8.4 (5.1, 13.5)	0%
**LYM**	103/mm3	2.2 (1.3, 3.4)	2.5 (1.5, 5.0)	2.6 (1.5, 3.2)	0%
**LYM %**	%	29.4 (22.1, 38.1)	27.5 (17.6, 37.4)	28.7 (20.4, 37.4)	0%
**MON %**	%	5.3 (2.6, 7.8)	5.6 (2.3, 8.7)	5.8 (1.9, 8.7)	0%
**GRA**	103/mm3	5.0 (3.3, 8.5)	6.2 (3.8, 9.0)	5.8 (3.3, 9.2)	0%
**GRA %**	%	66.2 (54.2, 74.4)	67.4 (56.2, 77.7)	65.7 (56.8, 77.5)	0%
**EOS %**	%	1.5 (0.5, 3.8)	1.5 (0.7, 8.0)	1.7 (0.7, 4.6)	0%
**Potassium**	mmol/l	3.2 (1.8, 4.8)	3.6 (2.5, 4.4)	3.4 (2.0, 4.0)	0%
**Sodium**	mmol/l	135.0 (131.0, 138.0)	135.0 (131.0, 138.0)	134.0 (130.0, 138.0)	2%
**Total calcium**	mmol/l	3.1 (2.9, 3.3)	3.1 (2.9, 3.3)	3.1 (3.0, 3.3)	0%
**CK**	U/l	244.5 (164.0, 420.0)	314.5 (214.0, 1217.0)	250.0 (184.0, 432.0)	0%

*WBC, white blood cells; LYM, absolute lymphocytes; LYM%, relative lymphocytes, MON, absolute monocytes; MON%, relative monocytes (MON%); EOS%, relative eosinophils; GRA, absolute granulocytes; GRA%, relative granulocytes; RBC, red blood cells; HBG, hemoglobin; MCV, mean corpuscular volume; MCHC, mean corpuscular hemoglobin concentration; MCH, mean corpuscular hemoglobin; RDW, red cell distribution width; PLT, absolute thrombocytes; MPV, mean platelet volume; CK, creatine kinase*.

According to qualification criteria, all horses were at least 8 years old and had successfully finished at least one 160 km ^***^ three star ride with a minimum average speed of 14 km/h in the past 24 months, and two 120 km ^**^ two start rides or higher in their career (15, 26). All horses were up to date on influenza vaccines as required by the FEI.

During the race, the average outside temperature was 18.9°C with 83% humidity. The track was mostly flat with no significant hills. The weather was partly cloudy with repeating rain showers and moderate wind speed.

The time between transportation, arrival at the venue and blood sampling differed among the horses ranging from 5 days before to the morning of the day before the start. All samples were drawn between 9:33 a.m. and 8:38 p.m.: 32.7% between 9 a.m. and 2 p.m. and 67.3% between 3 p.m. and 9 p.m. Before the sampling, each horse underwent a general examination in accordance with the endurance rules, excluding gait. Heartrate, skin turgor, mucosal membrane, back and girth, gut sounds and muscle turgor were recorded. Horses were not fastened. Weight was measured using standardized weight measuring tape.

The horses' presenters (rider, owner, groom, or team vet) were asked to report any treatment the horse had undergone within the last 7 days, especially fluid substitution directly prior to traveling or directly after arriving at the venue. For three horses this was unknown, so these animals were excluded from the statistical evaluation of the “infusion” variable.

Overall, 9.8 ml jugular venous blood was collected using the Braun vacutainer® system (Melsungen, Germany) with a 20-gauge-needle. Each horse was sampled with one 4 ml 13 × 75 mm PET hemogard K2 EDTA 1.8 mg/ml tube and one 4 ml 13 × 75 mm PET hemogard lithium heparin 17 IU/ml.

Two laboratory technicians conducted the blinded testing at the temporary laboratory set up at the venue. EDTA blood was used for hematology evaluation in the Scil VET ABC (Viernheim, Germany): white blood cells (WBC), absolute lymphocytes (LYM), relative lymphocytes (LYM%), absolute monocytes (MON), relative monocytes (MON%), relative eosinophils (EOS%), absolute granulocytes (GRA), relative granulocytes (GRA%), red blood cells (RBC), hemoglobin (HGB), mean corpuscular volume (MCV), mean corpuscular hemoglobin concentration (MCHC), mean corpuscular hemoglobin (MCH), red cell distribution width (RDW), absolute thrombocytes (PLT) and mean platelet volume (MPV) were measured. A lithium-heparin tube was used for the commercially available biochemistry panel in the VetScanVS2 (Abaxis, USA) with an Equine Profile rotor with full blood to measure sodium (Na+), potassium (K+), creatin kinase (CK) and calcium (Ca+2). Chloride was not included in the rotor and therefore could not be measured. According to requirements of Abaxis, Lithium heparin was spinned off at 4,000 g/4 min if hematocrit was >50% and then plasma was used for the Equine Profile Rotor. If hematocrit was <50% full blood was used.

Results were transferred directly from the laboratory machines to the Scil VIP Manager Software (Viernheim, Germany).

Elimination of 8-year-old horses was compared to the rest of the field using a Kaplan-Meier-Curve. As survival time the vetgate of elimination was used. Finishing horses were censored at vetgate 5. A *p*-value for the difference in survival between 8 and ≥9-year-old horses was computed using a log-rank test. The results data is available in Supplementary Table 1.

Horses were divided into three groups: finishing (18 horses), lame (20 horses), and metabolically (11 horses) eliminated athletes. All blood values were checked for normality using histograms and prior knowledge about distribution. Only CK had to be log-transformed to achieve a normal distribution. For statistical evaluation, we used multinomial logistic regression with finishing as reference vs. lameness or metabolic elimination (R package mgcv version 1.8-31) (27). We calculated univariable odds ratios with 95% confidence intervals. All possible confounders were identified using DAG's software DAGitty (28) and checked for association with exposure and outcome. Multivariable analyses were adjusted for the identified confounders of age, sex and weight. For all confounders, crude odds ratios are reported. Additionally, crude and adjusted odds ratios were calculated for infusion status. All *p*-values ≤ 0.05 were considered significant. All statistical analyses were performed using R Version 4.0.0 (R Foundation Vienna).

Breed was not considered to be a confounder because the variable “breed” only had a weak association with the race result (Cramer's *V* = 0.353) and most horses were either purebred or crossbred Arabians.

## Results

One hundred and thirty-one riders started in the race, 47 riders completed it. This equals to a completion rate of 35.87%, which is higher than the finisher rates of the world championships in 2014 (22.89%) and 2012 (35.37%) (29). No data for 2018 is available due to the ride being canceled.

Regarding the total race 21 horses (16.03%) were eliminated for metabolic issues, four riders retired (3.05%), three riders failed to complete a loop (2.29%), of which one suffered a catastrophic injury, and 56 horses (42.74%) were eliminated for lameness.

**Figure 1:**
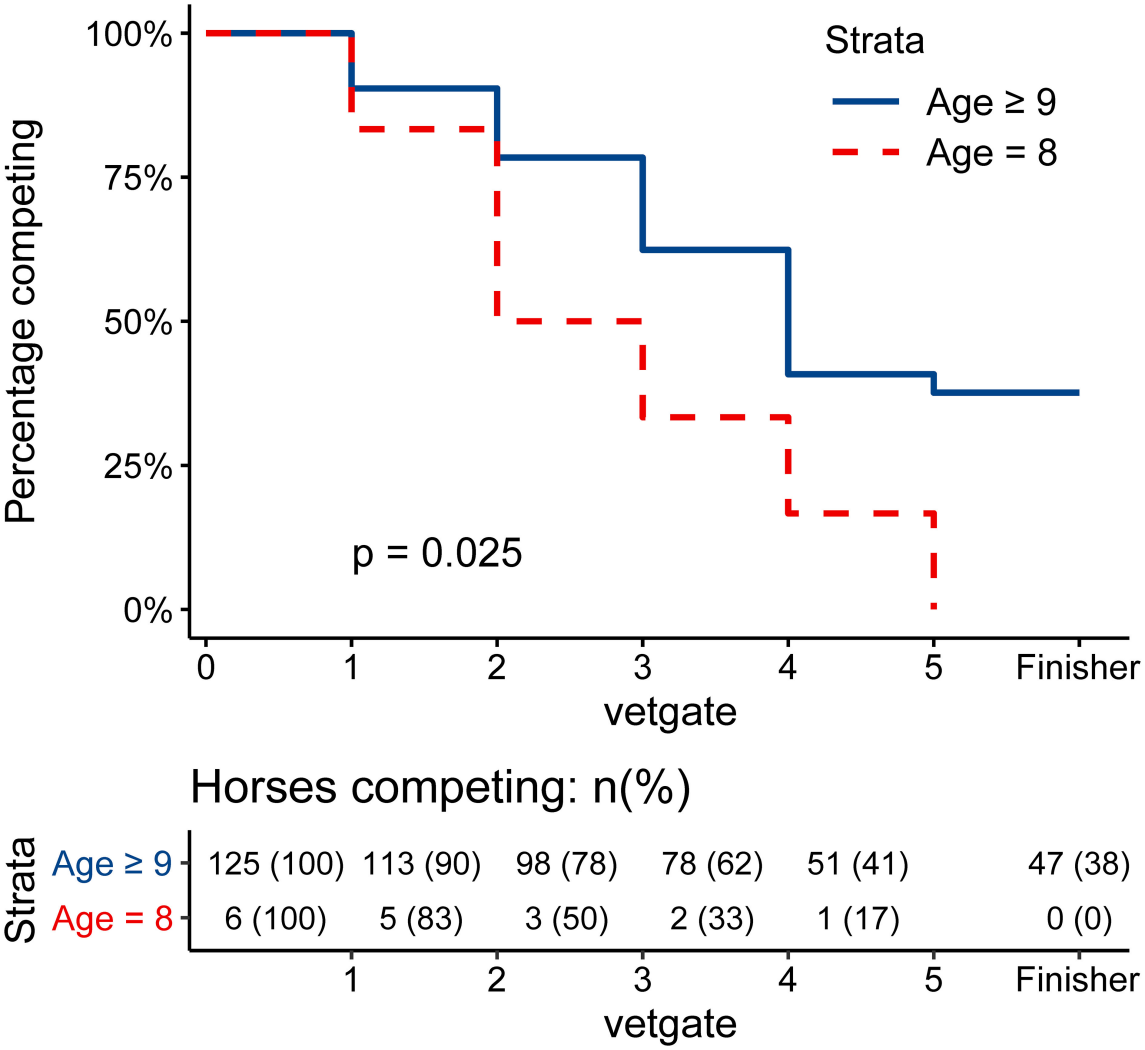
Survival Curve of the Elimination of 8-year-old horses vs. ≥9-year-old horses. Kaplan-Meier-Curve of the elimination of 8-year-old horses (red, dashed) and ≥9-year-old horses (blue, solid). The gate of elimination was used as the time point. Finishing horses were censored at gate 5 and are visually shown as a extra time point “Finisher.” *P*-value was calculated using a log-rank test. 8-year-old horses had a significantly higher hazard for elimination (*p* = 0.025) compared to ≥9-year-old horses.

None of the six 8-year-old horses of the 132 overall ride participants finished the race. The highest completion rate could be found among the 10-year-old horses (21 horses), where 57.1% finished the race. A similar percentage could be observed in the study population (Table 2). Survival analysis showed that 8-year-old horses had a significantly higher hazard for elimination (*p* = 0.025, see Figure 1) compared to ≥9-year-old horses.

**Table 2:** Dropout percentage by age.

**Age in years**	**All competing horses**		**Study population**	
	**Dropout (%)**	***n***	**Dropout (%)**	***n***
8	100	6	100	4
9	62.5	24	75	8
10	42.9	21	42.9	7
11	70	20	71.4	7
12	61.9	21	57.1	7
13	57.1	14	33.3	6
14	83.3	12	50	2
15	66.7	6	66.7	3
16	66.7	3	50	2
17	75	4	100	3

Forty-nine horses were included in the study. None of the horses showed abnormalities in the pre-sampling examination and all passed the pre-ride inspection. Of the 49 horses (23 mares/26 geldings), 11 horses (22.45%) were eliminated for metabolic issues, 20 horses (40.82%) were eliminated for lameness issues and 18 horses (36.73%) finished the race. The median age of the horses sampled was 12.0 (9.0, 16.0) for finishers, 11.0 (8.0, 17.0) for lame and 11.0 (8.0, 17.0) for metabolic horses.

The median weight of the horses was 405.0 (345.0, 440.0) kg for finishers, 402.0 (350.0, 450.0) kg for lame and 408.0 (380.0, 450.0) kg for metabolic horses.

All variables presented in the following section were adjusted for age, sex and weight. Only small changes in blood values were observed. None of the findings showed any indication of a pathological process.

**Figure 2:**
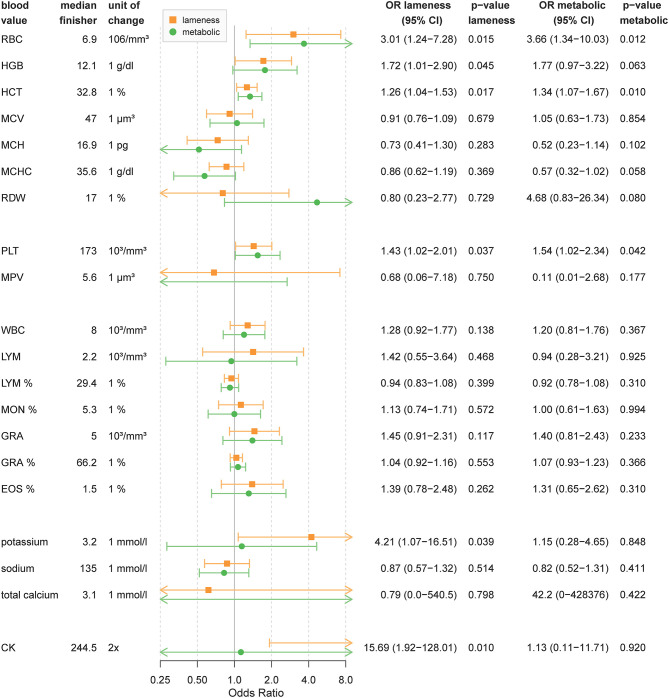
Blood value odds ratios for lameness or metabolic elimination. The figure shows odds ratios for elimination due to lameness or metabolic reasons depending on blood values based on 49 horses. Odds ratio are calculated for changes by one unit of change. OR above 1 mean that the odds of elimination increases with blood values higher than the finisher group. For OR below 1 the odds for elimination is higher for blood values lower than the finisher group. OR are shown as point estimates (circle or square) with 95% confidence intervals as error bars. Orange shows OR for lameness and green show OR for metabolic elimination. *P*-values below 0.05 can be considered significant. WBC, white blood cells; LYM, absolute lymphocytes; LYM%, relative lymphocytes; MON, absolute monocytes; MON%, relative monocytes (MON%); EOS%, relative eosinophils; GRA, absolute granulocytes; GRA%, relative granulocytes; RBC, red blood cells; HBG, hemoglobin; MCV, mean corpuscular volume; MCHC, mean corpuscular hemoglobin concentration; MCH, mean corpuscular hemoglobin; RDW, red cell distribution width; PLT, absolute thrombocytes; MPV, mean platelet volume; CK, creatine kinase.

The reference value for all following parameters is the median of finisher horses and can be seen in Figure 2.

As Figure 2 shows, for every 1% increase in hematocrit levels, horses had a 1.3-times higher chance of lameness and a 1.3-times higher chance of metabolic elimination. An increase of one unit of red blood cells (10^6^/mm3) resulted in a 3.0-times higher chance of lameness and a 3.7-times higher chance of metabolic elimination. Horses with an increased hemoglobin (g/dl) before the race had a 1.7-times higher chance of lameness and a 1.8-times higher chance for metabolic elimination per one unit increase. A higher amount of PLT increased the chance of elimination for lameness by 1.4 and metabolic reasons by 1.5 for every increase by 103/mm3.

Horses showing a significant elevation of CK before the competition had a 15.7-times higher chance of elimination for lameness for every doubling of the CK value. CK did not contribute to metabolic elimination. Pre-race elevation of potassium increased the chance of lameness elimination to 4.2-times per one unit compared to finishing horses but did not affect the chance of metabolic elimination.

Other measures of blood cell count or electrolytes only had a minor impact on the ride outcome (see Figure 2). Unadjusted OR are reported in Supplementary Table 2.

Eleven out of 18 horses of the finisher group (73.3%), seven out of 20 horses of the lameness group (35.0%) and 3 out of 11 horses of the metabolic group (27.3%) had received an infusion (intravenous administration of fluids) prior to or directly after transport. Horses that had received an infusion prior to or directly after transport had 82% reduced odds of lameness (OR = 0.181; 95% CI 0.038–0.851; *P* = 0.031) and 88% reduced odds (OR = 0.116; 95% CI 0.017–0.763; *P* = 0.025) of metabolic elimination after adjusting for age, sex and weight. Horses that had received fluid support traveled between 4 and 10 h without a recreational break. Horses traveling longer spent an overnight break midway so individual traveling times did not exceed 10 h according to the presenter. All horses with fluid substitution had received 5–10 L of 0.9% sodium chloride on the day of transportation. None of the horses receiving infusions after transport were sampled on the day of arrival.

As a sensitivity analysis, we adjusted the blood values using multinomial logistic regression for the infusion, which only resulted in a small decrease of the effect on the overall blood values. This is an indicator that pre- or post-transport infusion only explains a small portion of the observed value changes.

## Discussion

We analyzed race results, blood parameters and pre-race treatments of 49 horses competing in the World Championship of Endurance Riding. To our knowledge, this was the first time horses of an international endurance championship were examined.

We hypothesized that the change of different blood values prior to the ride would affect the chance of elimination. Differences among blood samples of the finishers, lame and metabolic eliminated horses in electrolytes and red blood cell count were expected. The study showed that reduced values of red blood cell count with lowered potassium had a positive impact on the outcome of the competition. Also, older horses with fluid substitution pre- or post-transportation had a higher possibility of completing the race.

The fact that sampling only took place before one race, multiple parameters were evaluated and there were only 49 participating horses must be kept in mind as limitations of this observational study. In addition, sampling times among the analyzed horses were not standardized as riders were not willing to change their regular routine due to the ride being a championship. Further information on the “infusion” variable—especially precise application times and individual transportation duration—were not provided by the presenters. A recent long-term evaluation showed a consistently decreasing starting age among horses since 2004 (7). The study indicated that the most reliable age for finishing is between 10 and 15, with chances of finishing increasing with every additional year of age. As heart rate variability also improves with age, it does not seem surprising that age may affect the chance of finishing (30). Numbers of starting and therefore included horses for analyzing the effect of age could not be influenced by the authors and were the highest in 10 years, except for Aachen 2006 (159 starting horses) and only minimally higher numbers in Euston Park 2012 (147 starting horses).

The tendency of having horses start at a younger age must be viewed very critically. Due to current FEI endurance regulations and international qualification modes, horses can start as early as 5 years of age and may enter international rides only 1 year later. Young horses are, therefore, at risk of not reaching the best performance age due to severe health issues resulting from early heavy and fast training. In the observed race, younger horses were more at risk of metabolic elimination than older horses (compared to lameness) and it is particularly notable that none of the 8-year-old horses finished the race. In addition, younger horses were also eliminated earlier from the race than older horses. Taking these findings into consideration, we recommend to reconsider the starting age for CEI ^***^ 160 km races and the minimum period between first qualification ride and first possible international start.

Further investigation of age influencing finisher rates, especially in combination with speed, is needed. Due to the ride being a championship, the atmosphere has been more competitive and therefore may have had encouraged riders to push the horses further than during normal rides (15). To make more general statements, measurements and analysis should be repeated under different conditions. The sampling included only one ride with moderate temperatures. The number of participating horses for the parameter age was evaluated for the 49 study horses and compared to the over-all number of 131 starting horses. Since these numbers were similar, we assume our results to be transferable. A retrospective evaluation of age and point of elimination of older championships could give useful information. Further investigation of the risk factor age of the authors is in progress.

Regarding the connection between weight and race results, Fielding et al. have already stipulated that the risk of elimination increases among breeds associated with a higher body mass index (18). According to our results, the weight of the horse may influence the chance of metabolic elimination. Although medians of weight in our study were similar among the tested groups, the interval for lame horses was slightly wider than for finisher horses. The weight interval for horses eliminated for metabolic reasons was notably higher and wider (380.0, 450.0 kg) and could not be explained by the difference in breeds.

Previous studies show a wide individual range for CK in finisher horses and CK alone does not serve as an indicator for successful race completion (31, 32). Therefore isolated CK values must always be evaluated in combination with clinical appearance and other laboratory parameters, such as myeloperoxidase or elastase or in combination with HCT as listed below (33). Elevated pre-competition CK values in our study population point to a significantly increased risk of elimination due to lameness. This might be caused by muscular damage (34) resulting from over-training, transportation stress (35), insufficient recovery breaks (36) before the ride or reduced renal elimination (37) due to dehydration (38). All these points influence potential muscle performance and, as a result, may lead to lameness elimination in the race (39).

The study data showed no major differences within the red blood cells and related parameters of lame and metabolic horses. However, these were significantly elevated compared to finishing horses. Values of RBC, PLT, HGB, and HCT in healthy horses can be affected by numerous factors, such as dilution, medical and supplemental support (e.g., iron), management, time of sampling, exercise prior to the ride, or individual horse traits. However, these factors have been consistent among all competing horses and are, therefore, not expected to play a role. Feeding has been identified as increasing HCT due to the loss of fluid in saliva and the gastrointestinal tract (40). According to the presenters, all of the horses had been offered hay continuously since arriving at the venue, which may have had a slight impact on our numbers.

Higher values of HGB and RBC in horses can also be caused by altitude training or the administration of erythropoietin during training (41, 42). Generally, elevated numbers of HGB and RBC are considered to have a positive impact on performance (41, 42). In contrast, low values for HCT, RBC, PLT, and HBG seemed to influence the outcome positively in our study. As altitude training has been reported to be a common measure in human athletes to increase HGB and RBC and to enhance performance (43–45), this would confirm doubts in human medicine that altitude training in elite compared to non-elite athletes turned out not to bring any further improvement (46, 47). An explanation for this quite surprising result might be that increased values of HGB, HCT, and RBC could be an indicator for a reduced hydration status. The hydration status being a possible cause of many problems leading to higher elimination rates is supported by Whiting (10). Insufficient hydration can be caused by stress during transportation (14), a lack of pre- or post-transportation fluid substitution, insufficient food and water intake, mismanagement in training, or uncomfortable accommodation. With regard to clinical relevance, the authors see pre-ride testing of hematology and CK as a useful tool to identify potential risk factors of elimination.

The study measured total calcium as this was included in the Equine Profile Rotor of Abaxis. For clinical use ionized calcium is preferred.

Chloride was not measured due to the use of commercially available testing kits on the venue. As chloride is one of the main electrolytes altered in endurance horses the missing measurement of chloride and the measurement of total calcium reflect limitations in the study design.

In order to compare hematology parameters, a standardized testing period before the ride is necessary. Sampling times could not be standardized due to the design of the study. As the FEI rules do not allow any needle insertion into the skin from 2 h before the pre-ride inspection until the end of the race, sampling of all horses 48 h before the race until 2 h before the pre-ride inspection would have been a proper time frame in order to standardize the procedure. However, as mentioned before, this was not possible as horses underwent individual routines the handlers did not want to adjust to a study protocol. HCT values of >40% and CK values of 470 U/l or higher may point to uncompensated problems. Trigo et al. use HCT > 52% and CK >12.6 ui/l as cut-off values, which seem too high according to our findings (24). A possible explanation for the significantly different figures may be the fact that all horses in this study count among the world's elite compared to more amateur horses in Trigos et al.'s work. Moreover, Trigo et al.'s study included different ride lengths in the evaluation (15^*^ one star rides, 9^**^ two star rides and 12^***^ three star rides on international FEI CEI level). Taking these numbers into consideration, the authors recommend withdrawing affected horses from the race. A potential treatment of these animals to improve blood values does not seem compatible with horse welfare, as the time frame would be too short for secure and sufficient recovery. However, more testing with greater numbers of horses is necessary to validate exact figures, possibly distinguishing between ride lengths as well as amateur vs. elite horses.

This study cannot determine whether finisher horses had been hydrated sufficiently or whether eliminated horses had been hydrated insufficiently prior to the race.

Our results show that low values of potassium were associated with a higher chance of finishing. According to our study, horses with higher potassium levels are more likely to be eliminated due to lameness. This confirms the findings of Fraipoint (48). The underlying clinical mechanism likely is an increased potassium excretion in the animal's urine, as McKeever et al. found in 1987. Only 5 weeks of training induced hypervolemia, reduced potassium plasma levels as well as osmotic renal clearance (49).

The potassium excretion is the result of an increased renal as well as fecal elimination, naturally associated with increased water loss (50). A decrease in hydration then leads to a reduced blood flow, followed by reduced muscle perfusion resulting in lameness and increased CK values. It is also possible that pre-ride increased plasma values of potassium prevent further muscle cell leakage of potassium leading to muscle fatigue (51, 52). Another result of increased potassium might be neuromuscular hyperexcitability, which could also result in elimination (53).

Since potassium plasma levels and HCT can be modified by training, both values in our study represent real alterations (49).

As all nations in our study used 0.9% sodium chloride for fluid substitution, lower values of potassium in finishers may also be the result of forced hydration due to the Na+-K+-ATPase.

However, our sensitivity analysis showed that the changes in blood values cannot solely be explained by pre- and post-transportation fluid substitution. It must be taken into consideration that this investigation might not be related to the common practice of infusion 1 day before the race. Time between sampling and last fluid administration differed and 6.1% of the study population could not give any information on fluid substitution. As feeding strategies were not assessed, it is also possible electrolyte values were influenced by nutritional supplementation (50).

Stress of transportation has been addressed as a major issue in other studies before and is highly likely to affect performance (54). According to Padalino et al. Arabians and crossbred Arabians are more susceptible to transportation stress (54). When arriving at the venue, horses' body reserves have been strained and time for compensation before the competition is short. Barton et al. found lactate to be lower in horses that had spent more time at the event venue compared to those staying there for shorter periods (55).

This raises the question of whether pre- or post-transport fluid substitution is an indicator for more professional management of a horse's metabolism, and whether it might be a proxy for advanced feeding, medical supplementation, transportation strategies and further optimization. However, we assume fluid substitution surrounding the transport including all related practices may have a positive effect on finishing.

## Conclusion

Our study for the first time assessed differences between finishers and eliminated endurance horses in a world championship. It indicates that more research on transportation stress of endurance horses, the questionable procedure of forced hydration and its effect on race outcome is necessary. An observational study fully capturing pre-race management and transport factors that could explain our observed blood value changes is needed.

We also recommend a randomized controlled trial for the effect of pre-race infusion on finishing as this is a common procedure in top-level endurance horses.

Retrospective evaluations of weight, especially age combined with speed and the duration of horses' endurance careers may lead to valuable insights in order to improve horse welfare and training in the future.

## Data Availability Statement

Aggregate participant data that underlie the results are available from the corresponding author on reasonable request. Results data is available in the supplement and under https://inside.fei.org/fei/disc/endurance/main-events/endurance-past.

## Ethics Statement

Ethical review and approval was not required for the animal study because the study was performed in accordance with EU Directive 2010/63/EU, the German Animal Welfare Legislation and the Guidelines of the Freie Universität Berlin on the protection of animals used for scientific purposes. Diagnostic blood collection is a common pre-ride procedure in elite equine athletes and constitutes a standard clinical veterinary practice. According to the mentioned legal regulations, the study cannot legally be classified as an animal experiment. Sampling therefore does not need any separate ethical approval, according to German legislation, as it is a common part of performance monitoring in endurance riding in accordance with good veterinary practice. The blood sampling was performed by trained veterinarians, while the owners or legal representatives (FEI licensed and accredited trainers) were present. Written informed consent for participation was not obtained from the owners because Information on the study was sent to all starting national federations in advance *via* email, which forwarded the information to their athletes. In addition, a social media post was placed to inform the endurance community. Verbal consent was obtained before any animal examination and sampling.

## Author Contributions

LB and HG have set up the hypothesis, experimental design, organized, conducted the experiments, analyzed, interpreted the results, and wrote and revised the mauscript. AB and AK analyzed, interpreted the results, and wrote and revised the manuscript. CW has set up the hypothesis, experimental design, organized, conducted the experiments, and wrote and revised the mauscript. All authors contributed to the article and approved the submitted version.

## Conflict of Interest

CW was employed by the company Laboklin GmbH & Co. KG. The remaining authors declare that the research was conducted in the absence of any commercial or financial relationships that could be construed as a potential conflict of interest.
