# Supplementary material for: Age and Hydration of Competing Horses Influence the Outcome of Elite 160 km Endurance Rides
Source: Front Vet Sci. 2021 May 14;8:668650. doi: 10.3389/fvets.2021.668650 (PMC8160124; doi:10.3389/fvets.2021.668650)
Supplement: Supplementary file 2 [file Table_2.pdf]

Supplement for “Age and Hydration of Competing Horses Influence the Outcome of Elite 160 km Endurance Rides”

Bollinger L, Bartel A, Küper A, Weber C, Gehlen H

DOI: 10.3389/fvets.2021.668650

Table S2 unadjusted crude OR

blood value	unit of change	OR lameness (95% CI)	p-value lameness	OR metabolic (95% CI)	p-value metabolic
**RBC**	$10^6/mm^3$	2.78 (1.18-6.57)	0.019	3.3 (1.25-8.74)	0.016
**HGB**	1 g/dl	1.67 (1.01-2.77)	0.045	1.7 (0.96-2.99)	0.068
**HCT**	1 %	1.23 (1.03-1.48)	0.024	1.3 (1.05-1.6)	0.015
**MCV**	1 μm^3	0.93 (0.64-1.36)	0.723	1.02 (0.66-1.58)	0.933
**MCH**	1 pg	0.81 (0.48-1.35)	0.419	0.62 (0.31-1.25)	0.184
**MCHC**	1 g/dl	0.9 (0.67-1.21)	0.489	0.68 (0.43-1.09)	0.109
**RDW**	1 %	0.72 (0.25-2.07)	0.536	2.14 (0.56-8.17)	0.267
**PLT**	$10^4/mm^3$	1.27 (0.95-1.68)	0.104	1.3 (0.92-1.83)	0.134
**MPV**	1 μm^3	0.78 (0.08-7.72)	0.832	0.13 (0.01-2.56)	0.178
**WBC**	$10^3/mm^3$	1.27 (0.92-1.74)	0.146	1.15 (0.79-1.66)	0.460
**LYM**	$10^3/mm^3$	1.49 (0.59-3.78)	0.402	0.96 (0.3-3)	0.938
**LYM %**	1 %	0.96 (0.85-1.09)	0.538	0.94 (0.81-1.09)	0.421
**MON %**	1 %	1.18 (0.81-1.71)	0.394	1.01 (0.66-1.56)	0.947
**GRA**	$10^3/mm^3$	1.39 (0.89-2.18)	0.151	1.29 (0.77-2.16)	0.337
**GRA %**	1 %	1.02 (0.91-1.13)	0.782	1.05 (0.92-1.19)	0.500
**EOS %**	1 %	1.21 (0.72-2.03)	0.464	1.19 (0.66-2.15)	0.554
**Potassium**	1 mmol/l	3.38 (1.04-10.96)	0.042	1.78 (0.51-6.24)	0.370
**Sodium**	1 mmol/l	0.96 (0.68-1.35)	0.798	0.95 (0.64-1.41)	0.800
**Total calcium**	1 mmol/l	0.61 (0-540.45)	0.888	98.97 (0.02-617161.25)	0.303
**CK**	2x	12.32 (1.59-95.43)	0.016	1.2 (0.12-11.6)	0.877
**Infusion**	yes=1/no=0	0.196 (0.045-0.850)	0.029	0.136 (0.023-0.786)	0.026
